# Aortic thrombus in a patient with myeloproliferative thrombocytosis, successfully treated by pharmaceutical therapy: a case report

**DOI:** 10.1186/1752-1947-4-219

**Published:** 2010-07-21

**Authors:** Hidesuke Yamamoto, Haruaki Nishimaki, Norikazu Imai, Masakazu Nitta, Osami Daimaru

**Affiliations:** 1 Department of Internal Medicine, Division of Hematology, Daiyukai General Hospital, 1-9-9 Sakura, Ichinomiya, Aichi 491-8551, Japan; 2 Department of Internal Medicine, Division of Hematology, Aichi Medical University School of Medicine, 21 Karimata, Yazako, Nagakute, Aichi 480-1195, Japan

## Abstract

**Introduction:**

Thrombosis in myeloproliferative thrombocytosis occurs usually in the microvessels and medium-sized arteries and veins and only rarely in the aorta. Aortic thrombosis is usually treated with thrombectomy. Reported here is a rare case that was treated pharmacologically.

**Case presentation:**

A 60-year-old Japanese woman presented with numbness of both lower extremities. Her platelet count was 1787 × 10^3^/μl. Through bone marrow examination, we diagnosed her condition as myelodysplastic and/or myeloproliferative disorder-unclassifiable. Abdominal ultrasonography and computed tomographic scan revealed aortic thrombosis. Her platelet count was controlled with hydroxyurea and ranimustine. Aspirin and ticlopidine improved the numbness in both lower limbs on the second day. Aortic thrombosis was not observed in a computed tomographic scan on the seventh day.

**Conclusion:**

For aortic thrombosis, surgical management is usually adopted, but pharmacological management is also an option because of its immediate curative effects.

## Introduction

Thrombocytosis is classified into myeloproliferative thrombocytosis and reactive thrombocytosis. In myeloproliferative thrombocytosis, there is always a risk of thrombosis. It is well known that thrombosis in myeloproliferative thrombocytosis occurs in the medium-sized arteries and veins and in the microvessels [1]. However, aortic thrombosis is rare [2,3]. We report a rare case of aortic thrombosis with myeloproliferative thrombocytosis. The main therapeutic method to resolve aortic thrombosis is a thrombectomy [4,5]. However, the aortic thrombus in our case was treated immediately pharmacologically. This case may serve as a reference for the choice of therapy for aortic thrombosis.

## Case presentation

A 60-year-old Japanese woman presented with numbness in both lower extremities. This symptom appeared four months prior to admission, and she experienced giddiness two months before. However, she had ignored these symptoms. Her medical history revealed that she had undergone an oophorectomy, and she had a uterine sarcoma at the age of 36 and mastitis at the age of 45. Her consciousness was clear but she had anaemic conjunctivae. Physical examination revealed that she was a well-nourished female with normal vital signs. She had no chest pain. Her liver and spleen were not palpable. Although she experienced numbness in both her lower limbs, the neurological findings were normal, and neither extremity was cold.

The patient had a white-blood-cell count of 2.74 × 10^3^/μl with 37% neutrophils, 62% lymphocytes and 1% monocytes. Her haemoglobin level was 7.8 g/dl and her platelet count was 1787 × 10^3^/μl. Examination of bone-marrow aspirate revealed normal cellular marrow, megakaryocytic hyperplasia and erythroid dysplasia. Her karyogram was normal.

**Figure 1:**
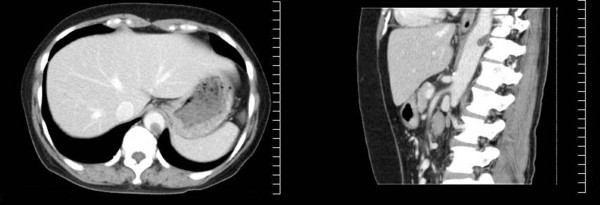
**Enhanced computed tomography scan before treatment**. A thrombus is observed in the lower-line aortic blood vessel at the level of the diaphragm.

Abdominal ultrasonography revealed in the aorta a moveable mass with a diameter of 1.5 cm. The interior of the mass had no blood supply. A computed tomographic scan of her abdomen revealed a circular thrombus in the descending aorta at the level of the diaphragm (Figure 1). Arteriosclerosis was not recognized at that point.

We diagnosed this case of thrombocytosis and anemia as myelodysplastic syndrome and/or myeloproliferative disorder-unclassifiable (MDS/MPD-U), and the aortic thrombus was thought to be associated with myeloproliferative thrombocytosis.

For myeloproliferative thrombocytosis, she was treated with 500 mg of hydroxyurea from the seventh day of her hospital stay. However, she was administered 1000 mg of hydroxyurea on the 16^th ^day because her platelet count increased to 2074 × 10^3^/μl. In addition, she was administered 50 mg of ranimustine on the 18^th ^day. The platelet count decreased to 274 × 10^3 ^/μl on the 39^th ^day, but it increased slowly thereafter. After administration of 1000 mg of hydroxyurea, her platelet count stabilized at approximately 1000 × 10^3^/μl. She was administered transfusions for anemia.

**Figure 2:**
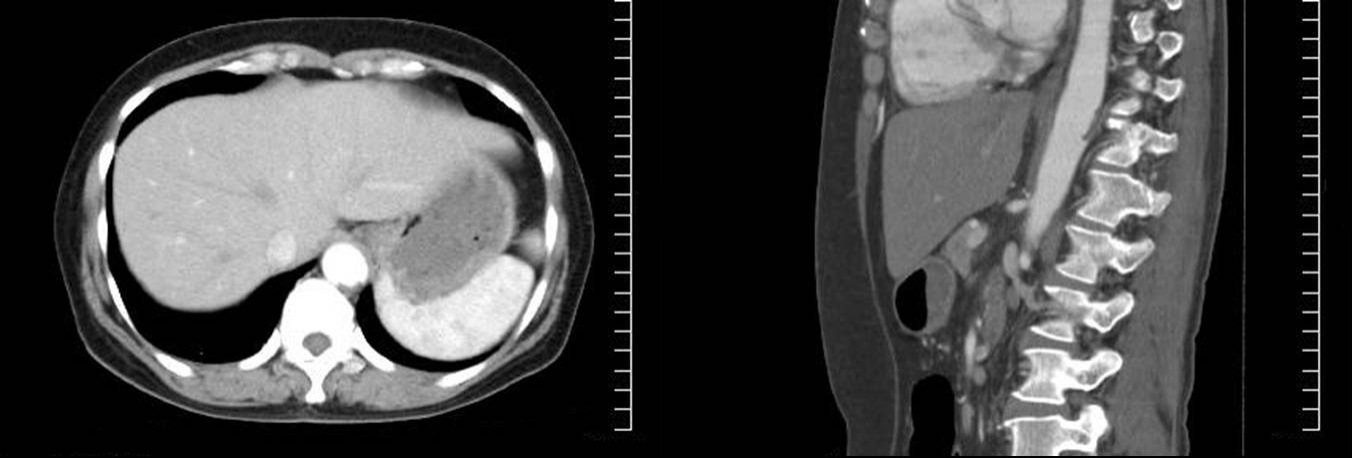
**Enhanced computed tomography scan after seven days of treatment**. The circular thrombus has disappeared. However, there is a low density area in the same spot, which was identified as a wall thrombosis.

Ticlopidine was administered to prevent the development of a thrombosis on the seventh hospital day, which was the same day treatment for myeloproliferative thrombocytosis was initiated. Aspirin (100 mg) was administered when an aortic thrombus was identified by computed tomographic scan on the 10^th ^day of her hospital stay. The numbness in both of her lower extremities disappeared within two days. Also, the aortic thrombus was not observed in the computed tomographic scan on the 17^th ^day of her hospital stay. She was administered aspirin after seven days (Figure 2). She has been without symptoms for more than one year.

## Discussion

MDS is haematological monoclonal disease characterized by dysplasia and ineffective haematopoiesis, which causes cytopenia [6]. Chronic myeloproliferative disorders (CMPD) are also haematological monoclonal diseases. However, they are characterized by an increase in the number of more than one type of myeloid lineage. There is a disease group that borders or overlaps both the MDS and CMPD disease groups. The World Health Organization (WHO) defined MDS/MPD in 2001 [7]. MDS/MPDs were classified as chronic myelomonocytic leukaemia, atypical chronic myeloid leukaemia, juvenile myelomonocytic leukaemia and myelodysplastic and/or myeloproliferative disorders-unclassifiable (MPD/MDS-U). MDS/MPD-U fails to meet the criteria for any of the specific MPD/MDS entities. On one hand, it is characterized by dysplasia and ineffective haematopoiesis, and on the other hand, it is characterized by an increase in the number of more than one type of myeloid lineage. A representative disorder of this group of diseases is refractory anaemia with ringed sideroblasts (MDS-RARS), wherein the platelet count is over 600 × 10^3^/μl [8]. The symptoms in this case are similar to those in the case of MDS-RARS and it satisfies the criteria for MPD/MDS; however, this case cannot precisely be diagnosed as MDS-RARS because Prussian blue reaction was not performed. The new WHO classification was published in September 2008 [9]. As for MDS/MPD, the name was changed to myelodysplastic and/or myeloproliferative neoplasm (MDS/MPN). According to the new classification, this case is a case of MDS/MPN-U.

It is well known that thrombosis occurs in medium-sized vessels and in microvessels in myeloproliferative thrombocytosis [1]. Of these thrombosis cases, including cases of cerebral infarction, transient cerebral ischaemic attack, retinal thrombosis, ischaemic heart disease, pulmonary infarction, hepatic artery and portal vein thrombosis, deep venous thrombosis and ischaemia of limbs, 11% to 25% are included in the myeloproliferative thrombocytosis group [10]. However, cases of aortic thrombosis are very rare among the myeloproliferative thrombocytosis cases [2,3]. Therefore, the stipulated treatment has not been established.

The prognosis of patients with myeloproliferative thrombocytosis is mainly determined by the onset of thrombosis; therefore, it is very important to control thrombosis [11]. Usually, in a case of aortic thrombosis, surgical management is adopted as the main therapy, and therapy in the form of medication is rare.

Johnson *et al *reported an essential thrombocythaemia-associated intra-aortic thrombus that was treated with an aortic thrombectomy, and they removed the thrombus, which consisted of platelets [4]. Sohn *et al *also described a case of surgical management [5]. This treatment is advantageous as surgical management prevents necrosis in the organ by an embolism in the peripheral arteries. However, aortic thrombectomy is a risky procedure. Ehrenfeld *et al *reported the case of a patient who died of shock after an aortic thrombectomy for thrombocytosis [12].

On the other hand, Fang *et al *reported a large aortic thrombus with essential thrombocythaemia that was treated with medication, and to the best of our knowledge, this is the only case in which this was done [13]. In this case, the symptoms disappeared within two days (similar to our case), and the disappearance of the thrombus was confirmed three weeks later by computed tomography. In our case, we confirmed the resolution of the thrombus eight days later. Because the symptoms disappeared within two days in both cases, the resolution of the thrombus might have occurred at similar times in both cases. If the thrombus can be dissolved early by pharmacological treatment, we believe that not only aortic thrombectomy but also pharmacological treatment can prevent necrosis in the organ. This implies that pharmacological treatment can be used as the primary treatment for this condition in the future.

Johnson *et al *identified a thrombus comprising platelets; this thrombus was termed a 'white clot' due to the characteristic thrombocythaemia [4]. Therefore, it is logical to treat patients with aspirin which is a cyclooxygenase (COX) inhibitor. Aspirin disrupts thromboxan A2 production by inhibiting COX-1 of the arachidonic acid cascade, and it exhibits antiplatelet action [14]. In particular, secondary aggregation, most of which result in the formation of large thrombi, such as an aortic thrombosis, is prevented. On the other hand, ticlopidine prevents platelet aggregation by specifically obstructing the adenosine diphosphate receptor P2Y12 [15]. In addition, it prevents shear-induced platelet aggregation. This case indicated that the combination of aspirin and ticlopidine can act synergistically because of their different mechanisms. In this case, there is a possibility that two types of antiplatelet agents contributed to early action.

This case showed that antiplatelet medications are effective for aortic thrombosis, and this case may serve as a reference in the choice of therapy for aortic thrombosis.

## Conclusion

We report an aortic thrombosis case with myeloproliferative thrombocytosis treated by medical management. Thus far, surgical management has been adopted for such cases. However, this report suggests that medical management is a safe and effective strategy for aortic thrombosis in the case of myeloproliferative thrombocytosis.

## Abbreviations

CMPD: chronic myeloproliferative disorder; COX: cyclooxygenase; MDS: myelodysplastic syndrome; MDS/MPD: myelodysplastic/myeloproliferative disorder; MDS/MPN: myelodysplastic/myeloproliferative neoplasm; RARS: refractory anaemia with ringed sideroblasts; U: unclassifiable; WHO: World Health Organization.

## Consent

Written informed consent was obtained from the patient for publication of this case report and any accompanying images. A copy of the written consent is available for review by the Editor-in-Chief of this journal.

## Competing interests

The authors declare that they have no competing interests.

## Authors' contributions

MN and OD analyzed and interpreted the patient data regarding the haematologic disease. NI and HN participated in the clinical evaluation of the patient. HY obtained written informed consent from the patient, carried out the literature search and drafted the manuscript. All the authors reviewed and approved the final manuscript.

## References

[B1] Watson KV Key N Vascular complications of essential thrombocythaemia: a link to cardiovascular risk factors Br J Haematol 1993 83 198 203 10.1111/j.1365-2141.1993.tb08272.x 8457467

[B2] Mitus AJ Schafer AI Thrombocytosis and thrombocythemia Hematol Oncol Clin North Am 1990 4 157 178 2155903

[B3] Valla D Casadevall N Lacombe C Varet B Goldwasser E Franco D Maillard JN Pariente EA Leporrier M Rueff B Muller O Benhamou JP Primary myeloproliferative disorder and hepatic vein thrombosis. A prospective study of erythroid colony formation in vitro in 20 patients with Budd-Chiari syndrome Ann Intern Med 1985 103 329 334 4026081 10.7326/0003-4819-103-3-329

[B4] Johnson M Gernsheimer T Johansen K Essential thrombocytosis: underemphasized cause of large-vessel thrombosis J Vasc Surg 1995 22 443 449 10.1016/S0741-5214(95)70013-7 7563405

[B5] Sohn V Arthurs Z Andersen C Starnes B Aortic thrombus due to essential thrombocytosis: strategies for medical and surgical management Ann Vasc Surg 2008 22 676 680 10.1016/j.avsg.2007.12.018 18513483

[B6] Brunning RD Bennett JM Frandrin G Myelodysplastic syndromes

[B7] World Health Organization Classification of Tumors Jaffe WS Pathology and Genetics, Tumor of Hematopoietic and Lymphoid Tissues 2001 Lyon: IARC Press 61 74

[B8] Vardiman JW Pierre R Bain B World Health Organization Classification of Tumors Jaffe WS Myelodysplastic/myeloproliferative diseases Pathology and Genetics, Tumor of Hematopoietic and Lymphoid Tissues 2001 Lyon: IARC Press 45 60

[B9] Gupta R Abdalla SH Bain BJ Thrombocytosis with sideroblastic erythropoiesis: a mixed myeloproliferative myelodysplastic syndrome Leuk Lymphoma 1999 34 615 619 10492088 10.3109/10428199909058492

[B10] Swerdlow SH Campo E Harris NL World Health Organization Classification of Tumours of Haematopoietic and Lymphoid Tissues 2008 Lyon: IARC Press

[B11] Harrison CN Platelets and thrombosis in myeloproliferative diseases Hematology Am Soc Hematol Educ Program 2005 409 415 16304412 10.1182/asheducation-2005.1.409

[B12] Cortelazzo S Viero P Finazzi G D'Emilio A Rodeghiero F Barbui T Incidence and risk factors for thrombotic complications in a historical cohort of 100 patients with essential thrombocythemia J Clin Oncol 1990 8 556 562 2307991 10.1200/JCO.1990.8.3.556

[B13] Ehrenfeld M Penchas S Eliakim M Thrombocytosis in rheumatoid arthritis. Recurrent arterial thromboembolism and death Ann Rheum Dis 1977 36 579 581 10.1136/ard.36.6.579 596952 PMC1000167

[B14] Fang M Agha S Lockridge L Lee R Cleary JP Mazur EM Medical management of a large aortic thrombus in a young woman with essential thrombocythemia Mayo Clin Proc 2001 76 427 431 10.4065/76.4.427 11322360

[B15] Goto S Understanding the mechanism of platelet thrombus formation under blood flow conditions and the effect of new antiplatelet agents Curr Vasc Pharmacol 2004 2 23 32 10.2174/1570161043476456 15320830

[B16] Gachet C ADP receptors of platelets and their inhibition Thromb Haemost 2001 86 222 232 11487010

